# Editorial: FDA-Approved Drug Repositioning for P-Glycoprotein Overexpressing Resistant Cancer

**DOI:** 10.3389/fonc.2021.632657

**Published:** 2021-03-17

**Authors:** Sungpil Yoon, Xiaoju Wang, Sompong Vongpunsawad, Gerard Tromp, Helena Kuivaniemi

**Affiliations:** ^1^ School of Pharmacy, Sungkyunkwan University, Suwon, South Korea; ^2^ Department of Pathology, University of Michigan, Ann Arbor, MI, United States; ^3^ Department of Pediatrics, Faculty of Medicine, Chulalongkorn University, Bangkok, Thailand; ^4^ Division of Molecular Biology and Human Genetics, Department of Biomedical Sciences, Stellenbosch University, Tygerberg, South Africa

**Keywords:** drug repositioning, resistant cancer, P-gp overexpresssion, MDR, anticancer drug, molecular targeting, lower toxicity, cancer stem cell

Anticancer drugs are an essential part of cancer treatment. Cancer cells can, however, develop resistance to these drugs by e.g., P-glycoprotein 1 (P-gp) overexpression or accumulation of mutations in the genes part of growth signaling pathways, apoptotic pathways, or repair system. Intrinsically, metastatic cancers, advanced-stage cancers, or stem cell-like cancers are usually drug-resistant and difficult to treat using current anticancer drugs. The overexpression of P-gp, also known as multidrug resistance protein 1 (MDR1) or ATP-binding cassette sub-family B member 1 (ABCB1), is one of the well-known mechanisms of resistance to anticancer drugs. Stem cell-like cancers often overexpress P-gp on their membranes, which results in inefficient treatment using the currently available anticancer drugs (1). It is, therefore, important to investigate novel therapeutic options to treat the P-gp overexpressing drug-resistant cancer cells. Identifying the mechanisms for targeting these cancers can overcome the inefficiencies of current anticancer drugs and lead to better outcomes for patients with P-gp overexpressing cancers.

Multiple P-gp inhibitors have been developed, however their toxicity in normal cells, particularly in combination with anticancer drugs, limits their utilities. Drug repositioning has been applied for the treatment of various diseases. It could lower the costs and speed up the process of developing drugs for the treatment of patients with drug-resistant cancer since repeating a large number of toxicity tests could be avoided (2). Food and Drug Administration (FDA) already has easily accessible data on the beneficial and adverse effects of a large number of drugs used in humans over a long period. Identifying existing FDA-approved drugs, which can be repositioned to target cancer cells that overexpress P-gp, can lead to better treatment in patients who develop resistance to anticancer drugs. As these drugs are already used in clinical settings, drug repositioning would offer an efficient method to address the urgent need for the pharmacological treatment of P-gp overexpressing drug-resistant cancer, allowing the approval of the treatment relatively rapidly. For example, metformin, a well-known drug used to treat type 2 diabetes mellitus, has anticancer effects and is clinically widely tested as a repositioned drug (3–7).

In this Research Topic issue, seven articles (three research and four review articles) present novel applications of drug repositioning to sensitize P-gp overexpressing resistant cancer cells and discuss better targeting strategies (Beklen et al.; Cho and Kim; Dahlmann et al.; Kim et al.; Lai et al.; Robinson and Tiriveedri; Seelig). These studies provide critical information needed for clinical trials, which could lead to therapeutic applications and incorporation of these drugs to treatment regimens in cancer.

Three research articles focus on drugs that target P-gp overexpressing resistant cancer cells (8–10). An article by Kim et al. describes novel findings with monotherapy for P-gp overexpressing resistant cancer cells. They used both *in vitro* and *in vivo* approache*s* to test 13 tyrosine kinase inhibitors, and demonstrated that crizotinib specifically targeted P-gp overexpressing cancer cells without P-gp inhibition. This finding indicates that crizotinib may potentially be used for resistant cancer patients, without the toxic effects of P-gp inhibition. Another study by Dahlmann et al. applied a combination therapy for metastatic colon cancer that is resistant to current anticancer drugs due to ABCB1 overexpression. Their findings suggest that co-treatment with lovastatin can increase the accumulation of the anticancer drug by transcriptionally reducing the ABCB1 expression and finally overcome P-gp overexpressing resistant cancers. The third research article by Beklen et al. investigated the transcriptional regulation of the MDR phenotype, including a new network-based approach. They identified a gene expression signature for colorectal cancer, which included the *ABCB1* gene and several other genes. The authors conclude that the best approach would be to identify drugs that inhibit P-gp *via* direct binding, but also reverse the gene expression profile of *ABCB1* and other co-repressed genes in various drug-resistant cancer types.

Two of the five review articles discuss drugs that target P-gp overexpressing resistant cancer cells (Cho and Kim; Lai et al.). Lai et al. identified 98 FDA-approved drugs with P-gp inhibitory activities by searching the DrugBank Database. The authors summarize clinical trials which used single or combination treatments of these drugs targeting specific cancer types (11). Another review article by Cho and Kim summarizes information available on various MDR-targeting drugs that inhibit P-gp overexpressing resistant cancer cells *via* different mechanisms, e.g., epithelial–mesenchymal transition, epigenetic modification, and factors such as hypoxia affecting tumor microenvironment (12). They found that these drugs inhibit specific signaling pathways for the reversal of MDR phenotype cancers. Additionally, considering that cancer stem cells develop throughout an individual’s lifetime, repositioned drugs with MDR-targeting ability are likely to reduce the incidence of cancer in susceptible individuals.

To develop highly specific inhibitors for targeting P-gp overexpressing resistant cancer, comprehensive studies on immune side-effects, body toxicity, and tumor-specific cytotoxicity of such inhibitors are necessary. The review articles by Robinson and Tiriveedri, and by Seelig, summarize the molecular mechanisms of P-gp (13, 14). These reviews emphasize that understanding the molecular function of P-gp is essential in developing specific P-gp inhibitors, as normal cells also have or increase P-gp expression on their membranes. Robinson and Tiriveedhi discuss the design of specific drugs for targeting P-gp overexpressing resistant cancer, because cancer-attacking immune cells in the tumor microenvironment also increase P-gp expression. They suggest that studying the 3D-crystal structure of P-gp with various mutations should be included in a drug-design strategy. In another review article, Seelig summarized the findings on the interaction of P-gp with the lipid sites of the substrate, drug-P-gp binding mechanisms, and the strength of P-gp interactions, to avoid toxicity in normal cells owing to P-gp targeting drugs. Considering that P-gp plays a role in eliminating oxidative waste to prevent cell apoptosis, the review also suggests drug-strategies for targeting the transcription of P-gp, by regulating its promoter activators.

Identifying therapeutic drug repositioning options (a single drug or a combination therapy) for targeting P-gp overexpressing resistant cancer cells could overcome the inefficiencies of the current cancer-targeting drugs and lead to better treatment options for patients with cancers that are resistant to the available drugs. The seven articles in this Research Topic highlight the different aspects of P-gp overexpressing resistant cancers and provide information on the development of improved treatment strategies. We hope the drug repositioning discussed in this Topic will facilitate initiation of clinical trials and lead to therapeutic application for P-gp overexpressing resistant cancers.

## Author Contributions

All authors listed have made a substantial, direct, and intellectual contribution to the work and approved the manuscript for publication.

## Funding

This work was supported by National Research Foundation of Korea (NRF) funded by the Ministry of Education (NRF-2019R1A2C2002923), and the Faculty of Medicine and Health Sciences, Stellenbosch University, South Africa.

## Conflict of Interest

The authors declare that the research was conducted in the absence of any commercial or financial relationships that could be construed as a potential conflict of interest.
